# The Hospitalization Costs of Diabetes and Hypertension Complications in Zimbabwe: Estimations and Correlations

**DOI:** 10.1155/2016/9754230

**Published:** 2016-06-15

**Authors:** Mutsa P. Mutowo, Paula K. Lorgelly, Michael Laxy, Andre M. N. Renzaho, John C. Mangwiro, Alice J. Owen

**Affiliations:** ^1^School of Public Health and Preventive Medicine, Monash University, Melbourne, VIC 3004, Australia; ^2^Centre for Health Economics, Monash University, Melbourne, VIC 3800, Australia; ^3^Helmholtz Zentrum München (GmbH), German Research Center for Environmental Health, Institute of Health Economics and Health Care Management (IGM), Ingolstädter Landstraße 1, 85764 Neuherberg, Germany; ^4^School of Social Science and Psychology, University of Western Sydney, Sydney, NSW 2751, Australia; ^5^Zimbabwe Diabetes Association, P.O. Box 1797, Harare, Zimbabwe

## Abstract

*Objective*. Treating complications associated with diabetes and hypertension imposes significant costs on health care systems. This study estimated the hospitalization costs for inpatients in a public hospital in Zimbabwe.* Methods*. The study was retrospective and utilized secondary data from medical records. Total hospitalization costs were estimated using generalized linear models.* Results*. The median cost and interquartile range (IQR) for patients with diabetes, $994 (385–1553) mean $1319 (95% CI: 981–1657), was higher than patients with hypertension, $759 (494–1147) mean $914 (95% CI: 825–1003). Female patients aged below 65 years with diabetes had the highest estimated mean costs ($1467 (95% CI: 1177–1828)). Wound care had the highest estimated mean cost of all procedures, $2884 (95% CI: 2004–4149) for patients with diabetes and $2239 (95% CI: 1589–3156) for patients with hypertension. Age below 65 years, medical procedures (amputation, wound care, dialysis, and physiotherapy), the presence of two or more comorbidities, and being prescribed two or more drugs were associated with significantly higher hospitalization costs.* Conclusion*. Our estimated costs could be used to evaluate and improve current inpatient treatment and management of patients with diabetes and hypertension and determine the most cost-effective interventions to prevent complications and comorbidities.

## 1. Introduction

The treatment of patients with complications associated with type 2 diabetes (T2DM) and hypertension (HTN) is costly due to the invasive investigations, hospitalization, and surgery that are required [1–3]. T2DM and HTN are two prevalent noncommunicable diseases (NCDs) in Sub-Saharan Africa (SSA) [2, 4], representing an economic burden estimated at 4 billion United States (US) dollars for diabetes in SSA and 3.7 trillion US dollars for HTN in low and middle income countries [2, 4]. Rates of T2DM and HTN in many urban areas of SSA are as high as, or higher than, those in most developed countries [5, 6], with T2DM and HTN predicted to overtake communicable diseases as the major cause of death by 2020 [7]. The prevalence of HTN in Africa (46%) is the highest in the world [8], while the prevalence of undiagnosed diabetes is much higher in low income countries (75.1%) compared to lower-middle and upper-middle income countries (46.0%) in SSA [9].

Antiretroviral therapy (ART) treatment for HIV (human immunodeficiency virus) has been linked to an increase in the numbers of HIV positive people developing metabolic syndrome, which includes T2DM and HTN [10]. Given the high prevalence of HIV in Zimbabwe, this could in turn cause a T2DM and HTN crisis in the future. As with HIV, T2DM and HTN are chronic diseases that require lifelong medical care and patient self-management and place a significant burden on health care systems. It is estimated that 5.9% of the Zimbabwean population live with T2DM and 34% live with HTN [11, 12]. In addition, there are a high number of cases of T2DM which remain undiagnosed [4], as well as high rates of uncontrolled HTN in patients with prescribed treatment [13]. Despite the prevalence of T2DM and HTN, funding for prevention programs for NCDs in Zimbabwe remains very low, with less than US$100,000 per year spent on programs nationwide from 2012 to 2013 [14]. Evidently NCDs accounted for 31% of total mortality the following year in 2014 [15].

T2DM and HTN pose a significant economic burden in Zimbabwe. The direct costs of both these chronic conditions to the patient, their family, and government include hospital and medication costs, while those who are of working age may be absent from work or have reduced productivity due to illness or disability.

In Zimbabwe there is increasing dependence on foreign donors for the majority of drug supply in the country [16, 17]. However, close to 90% of donor expenditure in Zimbabwe is used for communicable diseases and reproductive health and family planning programs [14]. In SSA, Zimbabwe has the third highest total cost of diabetes care per year for persons aged 20–79 years, after South Africa and Kenya [18]. Between 2001 and 2010 in Zimbabwe, the share of household out-of-pocket cost consumed by health expenditure in Zimbabwe increased from 36% to 52%, which is significantly higher than other countries in SSA [14, 18]. The prevention and treatment of T2DM and HTN are limited not only by financing of pharmacotherapies; another major constraint in low income countries, such as Zimbabwe, is that the majority of health care workers are not well trained in the detection of cases and treatment of patients [19].

Poor treatment and management of T2DM and HTN lead to intensified health care utilization and increased medical care costs and impose a significant societal burden [20]. Patient age, clinical and biochemical features, comorbidity of T2DM and HTN, presence of other comorbidities, and type of treatment have been found to significantly affect total medical costs [21–25]. T2DM and HTN incur medical expenditure not only through their direct treatment but also through treatment of sequelae such as cardiovascular (CVD), cerebrovascular (CVA), and renal diseases, complications which occur commonly in patients with T2DM and HTN [26]. Chronic complications require expensive medical procedures, such as limb amputation or renal dialysis, and significantly increase hospitalization costs and length of stay [27–30]. This burden is related not only to health care costs but also to indirect costs caused by loss of productivity from disability and premature mortality [18]. The prevention and management of complications are therefore a key factor in determining direct and indirect costs for patients with T2DM and hypertension.

There is limited literature on hospital costs for patients with T2DM and/or HTN in Zimbabwe. The analysis of hospitalization costs in patients, diagnosed with T2DM or HTN, is critical for informing the development of health policies and strategies that reduce disease burden in resource-limited settings. The purpose of this study is to examine and describe how medical treatment, health outcomes, and sociodemographic characteristics affect the direct hospital costs of inpatients, with T2DM or HTN as a primary diagnosis, who were admitted to Zimbabwe's largest tertiary referral hospital, Harare Central Hospital, during the period from 2012 to 2013. Harare Central Hospital is a public teaching hospital which provides medical care to around two-thirds of the urban population in Harare, the capital of Zimbabwe, as well as patients referred from district hospitals and clinics across the country. Harare Central Hospital is situated in a high density urban settlement [31] and predominately caters for people of lower socioeconomic status.

## 2. Methods

We retrospectively reviewed medical records of patients admitted to the hospital from 1 January 2012 to 31 December 2013 (two-year period) with at least one complication associated with T2DM or HTN, mainly, codes E10-E14 (diabetes complications), H54 (blindness, low vision), I10-I99 (hypertension and circulatory system), J18, J81, and J98 (respiratory disorders and pneumonia), N04-N28 (kidney diseases), and R00-R16, R29, and R41-R58 (symptoms and signs not elsewhere classified, symptoms and signs involving the nervous and musculoskeletal systems, gangrene), based on the International Statistics Classification Diagnostics and Health Problem tenth revision (ICD-10 codes). A complete list of the complications, comorbidities, and cause of death in the sample is found in supplementary Tables 1–3 in Supplementary Material available online at http://dx.doi.org/10.1155/2016/9754230.

All medical records are coded by a hospital number, and set of computer generated random hospital numbers was used to select admissions from the database of patients who had complications from 2012 to 2013. Paper medical records were retrieved from the records library by the resident hospital warden. As each patient medical record has a unique hospital number, data on the most recent admission during the two-year period were abstracted, thereby avoiding documenting multiple admissions of the same patient. The sample size for analysis of cost was based on the rule of at least 30 times the potential predictor variables [32]. The factors affecting treatment cost in the hospital were hypothesized to be patients' sociodemographic, clinical, and health outcome characteristics [33]. As there were 16 possible variables in the medical records (Table 1), a total of 500 medical records were selected and reviewed, and of those only 344 records, which included patients who had T2DM or HTN as a primary diagnosis, were used. There was no distinction between elective admissions and emergency admissions in the medical records. Data were collected on factors that could potentially impact hospitalization costs.

Our study hypothesized that patient sociodemographic characteristics, resource utilization (procedures and prescribed treatment), medical comorbidity, medical complication, and outcome (discharged or deceased) increase patient total hospital costs. Length of stay (LOS) has been found to be a highly correlated component of hospitalization costs, and focusing on processes of care and resource utilization of a better indicator of hospitalization costs [34]. LOS was therefore excluded in the model so as to assess other factors impacting costs. Patient sociodemographic characteristics examined were patient age, gender, marital status, number of dependents, residence (low or middle income), employment, and type of payment (insurance or cash). Data on patients' lifestyles were also extracted (smoked or consumed alcohol).

We adopted a “narrow” societal perspective whereby only hospital (health care provider) and patient out-of-pocket costs were included [35].

The unit costs of prescribed drugs and sundries and medical services were calculated employing a standard costing approach [36, 37]. The unit cost per medication was based on international drug prices from the Management for Sciences Health International Drug Price Indicator [38]. The cost of each patient's admission was individually calculated, with costs of the resources consumed daily summed up for the duration of admission. A bottom-up approach was used, which values utilization of data by type of care. This procedure yielded the total resource cost per individual as well as the overall cost for the study population (T2DM and HTN patients). Hospitalization costs documented in medical records had three main payment methods, out-of-pocket cash payments, medical insurance (pay out-of-pocket and claim a percentage of the money back later), and social security scheme (government paying), for 65+ years, epileptic, and psychiatric patients. A standardized data template that categorized resources into prescribed drugs, medical sundries utilized, medical procedures, length of stay, and the admission cost was used to extract data from the medical records. Resource utilizations were priced in US dollars, one of the many currencies used in Zimbabwe. Indirect costs, such as mortality, disability, and productivity losses resulting from lost work days, were not considered.

### 2.1. Statistical Analysis

User-written Stata code [39] which performs the Modified Park test was used to determine the most appropriate model specification within generalized linear models (GLM) with Poisson, negative binomial, or gamma variance functions [40]. The variance function accounted for variance increasing as medical spending increased [40]. Pearson's correlation test (for systematic bias in fit on raw scale), the Pregibon link test (linearity of response on the scale of estimation), and the modified Hosmer and Lemeshow test (systematic bias in fit on raw scale) were also performed for the GLM link [39]. Given the skewed distribution of cost data and nonzero costs and to avoid heteroscedasticity in simple least-squares models, we identified the GLM with a gamma distribution and a logarithmical link function as the appropriate model to study the effects of sociodemographic, clinical characteristics and medical treatment on direct hospital costs [41, 42]. Mean hospitalization costs were estimated by the model, firstly adjusted for diagnosis, age, gender, and outcome and then adjusted for diagnosis, outcome, and procedure.

As the purpose of our model was cost estimation, forward stepwise selection was used in the GLM to identify independent factors that could be predictive of hospital costs; a *p* < 0.05 was considered significant. Only those variables that were independently associated with hospitalization costs were included in the GLM model. Our model did not explore interaction effects between these independent variables. The beta coefficient from the GLM estimation was generated to indicate the relative increase in mean costs by increasing covariates by 1 unit. All results are presented as the mean ± standard deviation for normally distributed values, as the median with 25th percentile to 75th percentile range for nonparametric values, or as percentages. Statistical analysis was done using STATA 12 software (Stata Corp, College Station, TX, USA).

Permission and approval were granted from the Ministry of Health and Child Care in Zimbabwe (MoHCC), the Medical Research Council of Zimbabwe (MRCZ/B/1806), Harare Central Hospital Ethics Committee, and Monash University Human Research Ethics Committee. Data were collected retrospectively from July 2014 to September 2014.

## 3. Results 

### 3.1. Characteristics of the Study Population

The mean age of T2DM patients was 52.8 years (SD 20.1) with 35.9% being male; the mean age of HTN patients was 59.9 years (SD 19.6) with 36.4% being male. Patients with T2DM had a higher proportion (68.8%) of patients aged below 65 years than patients with HTN (50.7%). The median (IQR) length of stay and cost for patients with T2DM were higher (7 days (3–12), mean cost $1319 (95% CI: (981–1657)), median cost $994 (385–1553)) than those for patients with HTN (6 days (3.5–10), mean cost $914 (95% CI: (825–1003)), median cost $759 (494–1147)). The descriptive statistics of the characteristics of the patients are shown in Table 1.

### 3.2. Estimated Mean Costs for Procedures

The estimated mean hospitalization costs are displayed in Table 2 in two sections: firstly adjusted for diagnosis, age group, sex, and outcome and secondly hospitalizations costs adjusted for diagnosis and procedures. Total average costs were highest for female patients with T2DM below 65 years of age ($1467 (95% CI: 1177, 1828)). The estimated mean cost per procedure for T2DM patients was higher, regardless of outcome (discharge or death), than HTN patients. In-hospital mortality was higher in the female patient age group of 65+ years for patients with T2DM (40%) and HTN (33.7%). For male patients, in-hospital mortality was higher for patients with T2DM of 65+ years (60%); however, HTN patients aged below 65 years of age had a higher mortality rate (52.3%). Estimated mean costs per procedure for T2DM and HTN patients are listed in Table 2.

Sociodemographic and clinical variables independently associated with increased hospitalization costs (*p* < 0.05) included age (age group), employment (yes/no), systolic blood pressure, number of comorbidities, prescribed therapies, clinical procedures, and diagnosis (T2DM/HTN).  Table 3 displays the results from the forward stepwise GLM analysis for the hospitalization costs of patients with T2DM and HTN after adjusting for the set of covariates. Our analysis finds that prescribed therapies (*β* = 0.119, *p* < 0.001), wound care (*β* = 0.793, *p* < 0.001), dialysis (*β* = 1.232, *p* = 0.001), and amputation (*β* = 0.381, *p* < 0.036) are significantly associated with higher costs, whilst age group of 65+ years (*β* = −0.285, *p* < 0.001) was associated with lower costs. Although systolic blood pressure was found to be associated with cost, higher systolic blood pressure reduced costs by a very small amount (*β* = −0.002, *p* = 0.001). T2DM or HTN diagnosis did not have a significant impact on total cost in the multivariate analysis.

## 4. Discussion

We examined the use of health care resources and the associated hospitalization costs in a sample of patients with T2DM or HTN and at least one complication at Harare Central Hospital and found the economic burden of hospital cost for the patient to be considerable. Although it is expected that increased hospital costs are associated with patients aged over 65 years and not below 65 years, our results indicate that the cost of care was not uniformly distributed across patients, and female patients aged below 65 years with T2DM had higher hospitalization costs than patients with HTN. A younger age at diagnosis and female gender have been found to be associated with higher levels of medical spending for diabetes [43].

Patients discharged had higher costs than those who died in hospital; however, factoring in the indirect mortality cost would result in a higher out-of-pocket expense due to the cost of death (funeral expense from hospital) for the patients' immediate family. A local funeral service company estimated the funeral expense (average cost of death) to be US$1,634, with the cost including transport costs of the body from Harare Central Hospital, body embalming, mortuary expense, and coffin and burial expenses [44]. Patients who underwent amputation and blood transfusion procedures had 100% in-hospital mortality. The high in-hospital mortality rates highlight possible barriers to health care access, late presentation of patients with complications, and the inadequate response and facilities of the hospital, which is struggling to provide affordable and optimal care for T2DM and HTN in Zimbabwe.

Our model found that age below 65 years, increasing prescribed drug therapies (2 or more drugs), procedures (specifically amputation, wound care, dialysis, and physiotherapy), and the presence of two or more comorbidities were significantly associated with higher hospitalization costs.

Our study revealed that age below 65 years was associated with increased hospital costs, contrary to previous studies that have reported older age being associated with higher costs [45, 46].

The age of onset of T2DM in SSA is decreasing, with peak occurrence between the ages of 20 and 44 years, 40 years lower than the peak age of occurrence in high income countries [47, 48]. It was estimated that on average each additional year with diabetes increases annual medical expenditures by 158 US dollars (standard error = $38) [49]. These two factors are likely explanations to the higher costs of hospitalization associated with being aged below 65 years. The presence of two or more comorbidities and prescription of two or more drugs were found to be associated with higher costs in our study, likely to reflect more intensive treatment and surgical intervention required to manage comorbid medical conditions such as comorbid T2DM and HTN, peripheral arterial disease, and communicable diseases such as HIV, tuberculosis (TB), and malaria [50]. A study in Tanzania found over 10% of patients diagnosed with malaria had precoma or coma induced by uncontrolled diabetes on admission [51], while another study in Nigeria found that the mean total cost of treatment for patients with HTN was significantly higher for those with comorbidity [52]. Prevention of comorbidity in patients, through implementation of primary prevention strategies, including lifestyle modifications, could therefore reduce drug treatments and costs and possibly improve hospital outcomes.

Hospitalization, wound care treatment materials, and nursing time requirements for wound dress changes have been found to be the main cost drivers for wound care in the health care system, accounting for almost 4% of total health system costs. These findings support our results of wound care being associated with higher costs for patients [53, 54]. Roughly 12% of all diabetic patients in SSA have foot ulcers, with up to 7% of hospitalized diabetic patients requiring amputation [55]. Amputations due to ulcers may escalate hospital costs through extended length of stay, operations, excessive morbidity, and even mortality [56]. Process measures such as diabetic foot off-loading, venous ulcer compression, or vascular screening are known measures for preventing and reducing the complications and costs of amputations and wound care management [57].

Given the rehabilitation required for complications such as stroke and cardiac failure, it is expected that physiotherapy would be associated with higher costs for patients in our sample. A cost of treatment analysis in Nigeria revealed that 46% ($734) of total direct costs were spent on physiotherapy after a stroke event, less than half of the total cost estimated in our study [58]. Dialysis was associated with higher costs, a finding similar to other countries in SSA, where only 20% of patients could afford dialysis three times a week and 70% could afford it only once a week [59]. In addition to this, no kidney transplant operations are available in Zimbabwe and Harare Central Hospital has a limited number of dialysis machines, possibly explaining the high mortality rate due to renal failure for both patients with T2DM and patients with HTN.

### 4.1. Study Limitations

Limitations of our study should be noted. First, GLM models with a log link force estimates of the incremental effect of each independent variable on cost to vary with the levels of the other independent variables in the model [60]. Our calculations did not consider the possibility that interaction effects between independent variables might increase or decrease cost estimates. In addition to this, the individual standard errors from the GLM output do not capture the full conditional uncertainty of a given interacting independent variable [61]. Second, we relied on the accuracy and completeness of patient medical records; however, as no distinction was made between elective and emergency admissions, it is difficult to ascertain factors that affect cost between the two. Third, this was a cross-sectional study and our sample did not consist of individually matched patients without T2DM and/or HTN complications or list previous expenses for patients. We therefore were not able to isolate incremental costs attributable to T2DM, HTN, or their related complications [62]. The cross-sectional design did not give insight into causal associations between costs, their associated factors, and the quality of care provided, which would affect outcomes. Fourth, those with low costs might come earlier, but more often, to the hospital. As we only have total costs of one admission, this might bias the expected overall inpatient costs of the diseases and complications.

Fifth, we did not enter each comorbidity separately and only analyzed the presence of comorbidity not the specifics of each. It is possible that certain comorbidities significantly contribute to direct medical costs more than others. Finally, the costs in our model reflect direct medical costs and do not take into account indirect costs (e.g., mortality, family care-takers, and reduced or lost productivity).

## 5. Conclusion

The present research provides a general estimate of the costs of complications for patients with T2DM and HTN in Zimbabwe and can serve as a resource for providers, payers, and researchers aiming to better understand the overall impact of these conditions. The Sustainable Development Goals (SDGs) have the target of reducing premature mortality from NCDs by one-third through prevention, treatment, and promoting mental health and well-being [63]. Prevention and management of T2DM and HTN are yet to be prioritized in Zimbabwe. From the results of this study, it is suggested that health care providers and health policymakers may need to focus on the factors associated with an increase in hospitalization cost and interventions such as disease management for patients to delay the progression of comorbidities or complications that may reduce health care expenditure. NCD programs and campaigns are urgently needed to raise awareness, educate the masses as a prevention strategy, and improve treatment and management of patients with T2DM and HTN. A lower financial burden on individuals by increasing public health funding could increase treatment compliance, improve management of T2DM and HTN, and reduce comorbidity and complications, thus reducing the burden on the struggling health care system.

## Supplementary Material

A list of the comorbid conditions of patients with type 2 diabetes and patients with hypertension are provided in Supplementary Table 1. The associated complications of patients with diabetes and hypertension are listed in Supplementary Table 2. The primary causes of death listed in the patients' medical records are listed in Supplementary Table 3.

## Figures and Tables

**Table 1:** Descriptive statistics of T2DM and HTN patients admitted to Harare Central Hospital.

Characteristics	T2DM (*n* = 64)	HTN (*n* = 280)	All patients (*n* = 344)
Age (mean ± SD)	52.8 ± 20.1	59.9 ± 19.6	58.6 ± 19.8
<65 years (%)	44 (68.8)	142 (50.7)	186 (54.1)
65+ years (%)	20 (31.3)	138 (49.3)	158 (45.9)
Male (%)	23 (35.9)	102 (36.4)	125 (36.3)
Married (%)	36 (56.3)	168 (60.0)	204 (59.3)
Dependents (median, IQR)	2 (0–3)	1 (0–2)	1 (0–2)
Employed (%)	16 (25.0)	27 (9.6)	43 (12.5)
Low income urban residence (%)	58 (90.6)	254 (90.7)	312 (90.7)
Family history (yes, %)	6 (9.4)	6 (2.3)	12 (3.7)
Smoking (yes, %)	5 (7.8)	5 (1.8)	10 (2.9)
Alcohol (yes, %)	5 (7.8)	15 (5.4)	20 (5.8)
Blood pressure > 140/90 mmHg	19 (29.7)	104 (37.1)	123 (35.8)
Prescribed therapies			
Monotherapy	4 (6.3)	7 (2.5)	11 (3.2)
Double therapy	3 (4.7)	34 (12.2)	37 (10.8)
3+ prescribed drugs	57 (89.0)	237 (85.3)	294 (85.5)
Comorbidity			
No comorbidity (%)	30 (46.9)	158 (56.4)	188 (54.7)
1 comorbidity (%)	30 (46.9)	101 (36.1)	131 (38.1)
2+ comorbidities (%)	4 (6.3)	21 (7.5)	25 (7.3)
Complications			
1 complication	56 (87.5)	235 (83.9)	287 (84.6)
2+ complications	8 (12.5)	45 (16.1)	53 (15.4)
Length of stay days			
Median (IQR)	7 (3–12)	6 (3.5–10)	6 (3–10)
In-hospital mortality (%)	24 (37.5)	91 (32.5)	115 (33.4)
Hospitalization costs (USD)^*∗*^			
Average cost per admission (95% CI)^*∗*^	1319 (981–1657)	914 (825–1003)	990 (893–1087)
(minimum–maximum)	(121–7153)	(113–6606)	(113–7153)
Median cost per admission (IQR)^*∗*^	994 (385–1553)	759 (494–1147)	780 (483–1179)
Total patients < 65 years costs	48,627	141,267	189,894
Total social security cost	20,904	129,675	150,579
Payment method			
Out-of-pocket cash	19 (29.7)	140 (50)	158 (45.9)
State paying (social security scheme)^∧^	43 (67.2)	139 (49.6)	183 (53.2)
Medical insurance	2 (3.1)	1 (0.4)	3 (0.9)

^*∗*^Costs from patient perspective; IQR, interquartile range; SD, standard deviation; costs in USD, United States dollars; CVD, cardiovascular disease; CCF, congestive cardiac failure; CVA, cerebrovascular accident.  ^∧^Social security exempts payment for patients 65+ years and patients with epilepsy and mental illness.

**Table 2:** Estimated mean hospitalization costs for patients with T2DM and HTN.

Patient characteristics	T2DM diagnosis		Hypertension diagnosis	
	Discharged	Died	Discharged	Died
	Mean cost (95% CI)	Mean cost (95% CI)	Mean cost (95% CI)	Mean cost (95% CI)
	*n* (% discharged)	*n* (% died)	*n* (% discharged)	*n* (% died)
Women				
<65 years of age	$1467 (1177, 1828)	$1308 (1011, 1691)	$1124 (961, 1314)	$1002 (813, 1234)
	18 (69.2)	8 (30.8)	79 (80.6)	19 (19.4)
>65 years of age	$1037 (797, 1349)	$924 (695, 1230)	$794 (676, 933)	$708 (580, 864)
	9 (60)	6 (40)	53 (66.3)	27 (33.7)

Men				
<65 years of age	$1443 (1116, 1866)	$1286 (984, 1681)	$1105 (910, 1343)	$985 (797, 1218)
	11 (61.1)	7 (38.9)	21 (47.7)	23 (52.3)
>65 years of age	$1020 (761, 1366)	$909 (679, 1217)	$781 (643, 949)	$696 (571, 849)
	2 (40)	3 (60)	36 (62.1)	22 (37.9)

Procedures				
No procedure	$1114 (899, 1380)	$868 (677, 1113)	$865 (770, 972)	$674 (571, 797)
	31 (64.6)	17 (35.4)	137 (69.2)	61 (30.8)
Dialysis	$1347 (1095, 1657)	$1050 (833, 1325)	$1046 (929, 1178)	$816 (700, 950)
	1 (50)	1 (50)	8 (53.3)	7 (46.7)
Physiotherapy	$1630 (1303, 2038)	$1270 (1003, 1610)	$1266 (1081, 1482)	$987 (832, 1170)
	1 (50)	1 (50)	41 (70.7)	17 (29.3)
Amputation	—	$1537 (1183, 1996)	—	$1193 (965, 1475)
		2 (100)		2 (100)
Blood transfusion	—	$1859 (1375, 2512)	—	$1443 (1106, 1884)
		2 (100)		2 (100)
Wound care	$2884 (2004, 4149)	$2248 (1582, 3194)	$2239 (1589, 3156)	$1746 (1259, 2422)
	6 (85.7)	1 (14.3)	3 (60)	2 (40)

GLM: estimated costs from GLM model for hospitalization costs adjusted for age, for sex, for outcome, and then separately for procedures. Costs in USD, United States dollars.

**Table 3:** Forward stepwise regression analysis of variables predictive of hospitalization cost (*n* = 344).

Variables^*∗*^	Coefficient	Std. error	*p* value
(Intercept)	5.59	0.27	

Age (reference: <65 years of age)			<0.001
65+ years	−0.29	0.08	

Prescribed therapies			
3+ therapies	1.66	0.23	<0.001
2 therapies	1.24	0.25	<0.001

Procedures			
Wound care	0.78	0.20	<0.001
Amputation	1.16	0.35	0.001
Dialysis	0.65	0.18	<0.001
Physiotherapy	0.22	0.10	0.026

Comorbidity			
2+ comorbidities	0.32	0.14	0.026

Systolic blood pressure	−0.002	0.001	0.031

Data were analyzed with a generalized linear model (GLM), gamma family, and log link. ^*∗*^Data are shown only for variables that remained in the final model with a significance threshold of 0.05.
