# Supplementary material for: The Hospitalization Costs of Diabetes and Hypertension Complications in Zimbabwe: Estimations and Correlations
Source: J Diabetes Res. 2016 Jun 15;2016:9754230. doi: 10.1155/2016/9754230 (PMC4925986; doi:10.1155/2016/9754230)
Supplement: Supplementary file 1 — A list of the comorbid conditions of patients with type 2 diabetes and patients with hypertension are provided in Supplementary Table 1. The associated complications of patients with diabetes and hypertension are listed in Supplementary Table 2. The primary causes of death listed in the patients' medical records are listed in Supplementary Table 3. [file 9754230.f1.pdf]

Supplementary Tables

Table 1 List of Comorbid conditions in the sample

Comorbidity	
1	Hypertension
2	Diabetes Mellitus
3	HIV
4	Cardiovascular disease
5	Kidney disease
6	Respiratory disease
7	Gastrointestinal system disease
8	Cancer
9	Septicemia
10	Chronic liver disease
11	Anemia
12	Meningitis
13	Nutritional deficiency
14	Epilepsy
15	Cellulitis
16	Deep vein thrombosis
17	Arthritis
18	Herb/drug poisoning
19	Gangrene
20	Epistaxis
21	Endocrine disorder
22	Urinary tract infection

Table 2 Complications of patients in the sample

ICD-10 code	Complications
E10-E14	Diabetes Complications
H54	Blindness, low vision
I10-I99	Hypertension and circulatory system
J18, J81, J98	Respiratory and pneumonia
N04-N28	Kidney diseases
R00-R16, R29, R41-R58	Symptoms and signs not elsewhere classified, Symptoms and Signs Involving the Nervous and Musculoskeletal Systems, gangrene

ICD-10 – International classification of diseases

Table 3 Primary cause of death listed in medical record

	Cause of death listed
1	Heart failure
2	Stroke
3	Renal Failure
4	Diabetic complications
5	Hypertension
6	Pneumonia
7	Malaria
8	Peripheral Vascular disease
9	Rheumatic heart disease
10	Chronic diarrhea
11	Tuberculosis
12	Gastric malignancy
13	Cellulitis
14	Sepsis
15	Gastrointestinal disease
16	Cancer
17	CVD
18	HIV/AIDS
19	Anemia
20	Liver failure
21	Malaria
